# Autophagy-related genes affect the survival of multiple myeloma patients depending on chromosomal abnormality

**DOI:** 10.2478/abm-2022-0028

**Published:** 2023-06-16

**Authors:** Gizem Ayna Duran, Yasemin Benderli Cihan

**Affiliations:** Department of Biomedical Engineering, Faculty of Engineering, Izmir University of Economics, Balçova, İzmir 35330, Turkey; Department of Radiation Oncology, Kayseri City Education and Research Hospital, Kocasinan, Kayseri 38080, Turkey

**Keywords:** autophagy, chromosomal abnormality, gene, multiple myeloma, survival

## Abstract

**Background:**

Targeting autophagy at gene level may be promising in multiple myeloma (MM) treatment depending on chromosomal abnormality (ABN) status.

**Objectives:**

We aimed to investigate the role of ABN on survival of MM patients and to identify prognosis related autophagy-related genes (ARGs) for patients with or without ABN.

**Methods:**

Gene intensity values of 222 ARG for 548 MM patients were obtained from the Affymetrix Human Genome U133 Plus 2.0 Array (GPL570) platform containing 54,675 probes (GSE24080). A dataset containing data from 1576 MM patients with 1q21 amplification (GSE4204, GSE4452, GSE4581, and GSE2658) was used for validation. Survival analysis of the patients was analyzed using univariate and multivariate Cox regression method with the help of R3.53 programming language and Kaplan–Meier graphics were created. The Gene Ontology enRIchmentanaLysis and visuaLizAtion (GOrilla) tool was used to define the related biological processes and pathways.

**Results:**

The overall survival (OS) and event-free survival (EFS) in all MM patients were strongly influenced by ABN. In the group of patients with ABN, 41 ARGs were found to be important in prognosis, whereas in the group of patients without ABN, 13 ARGs were found to be important in prognosis. *CDKN1A*, *FKBP1B*, *FOXO3*, and *NCKAP1* ARGs were commonly significant in both groups and found to be survival triggering.

**Conclusions:**

The classification of MM patients according to the absence or presence of ABN is important in the determination of survival status. Detection of survival related ARGs in patients with chromosomal anomalies may be a new therapeutic target in treatment.

Autophagy is lysosomal degradation of cellular proteins by autophagic vacuoles. It is a self-digesting catabolic process that occurs in all eukaryotes and plays an important role in maintaining cellular homeostasis. Autophagy develops in cells that lack nutrients, oxygen, or growth factors. Although it has been known for many years, its mechanism has not been fully explained [1]. Studies have reported that autophagy plays a role in metabolism, cancer, neurodegenerative diseases, infections, morphogenesis, aging, cell death, and the immune system [1,2,3,4].

The role and regulation of autophagy in cancer is quite complex and has different effects according to different stages of cancer formation. It can suppress the formation of cancer cells by breaking down damaged and potentially dangerous components within the cell in the early stages of cancer formation. However, in the advanced stages of cancer, it causes the growth of cancer cells by contributing to the survival of the cell, by creating resistance against stress conditions such as starvation [2, 5,6,7]. In recent years, it has been shown that autophagy can contribute to cancer cell formation in the early stages of cancer, while it has also been shown that cancer cell growth can be suppressed by triggering autophagy in later stages [8]. As can be seen from these contradictory results, although the role of autophagy in cancer types and stages has not been fully clarified yet, studies have shown that targeting autophagy at the gene level has promising effects in the treatment of cancers. According to recent studies, it has been shown that autophagy may be one of the important mechanisms in the development of resistance to chemotherapy, as well as hormone and radiation therapy [2, 3, 5].

Autophagy is tightly regulated in the body by tumor suppressor genes and oncogenes. To date, 222 autophagy-related genes (ARGs) have been identified that contribute directly or indirectly to the autophagic process. These ARGs are known to play a role in a variety of diseases, including cancers. It is stated that the expressions of some molecules associated with autophagy differ in different cancer types. It is estimated that ARGs also play a role in multiple myeloma (MM) [9,10,11]. Studies are ongoing to identify these genes and to clarify their important roles in MM.

Today, more specific and less toxic treatment options are needed in MM, as in all cancers. For this reason, it is thought that the autophagy pathway that can contribute to the development of new treatment approaches in MM is an important target.

In this study, an attempt has been made to determine the prognosis-related ARGs in MM patients with and without chromosomal abnormalities (ABN). To our knowledge, our study is the first to detect and group the prognosis-related ARGs into MM patient groups with and without chromosomal abnormality (ABN). To sum up, we have successfully shown that the hazardous and survival-triggering genes differ quite substantially in the presence and absence of ABN, and it is important to optimize combination cancer therapies on this platform.

## Material and methods

Ethics committee approval was not necessary for this study because publicly open information from the Gene Expression Omnibus (GEO) database (ncbi.nlm.nih.gov/geo/) was used.

### Data acquisition and data processing

A total of 548 patients diagnosed with MM between 2000 and 2008 were included in this study. Although the data included 559 patients’ gene intensity values, the number of patients whose data was used in the study was reduced to 548 due to missing values for some variables. The data were accessed using accession number GSE24080, which is an output of the MAQC-II Project: MM dataset. On the other hand, the dataset does not include detailed information on the type of ABN. Instead, it provides the absence or presence of ABN in MM patients. Information on the demographic characteristics of the patients and microarray expression profiles were obtained from the Gene Expression Omnibus (GEO) database (ncbi.nlm.nih.gov/geo/). Age, gender, race, treatment protocol, and ABN, as well as follow-up time and survival time (EFS and overall survival [OS]) of the patients, were obtained from this database. Additionally, cDNA microarray data of ARG, amounting to a total of 222, were obtained using the human autophagy database (HADb; autophagy.lu/). Gene intensity values of 222 ARG (MAS5 log2 intensity values of the genes) for MM patients were obtained from the [HG-U133_Plus_2] Affymetrix Human Genome U133 Plus 2.0 Array (GPL570) platform containing 54,675 probes (Affymetrix; Thermo Fisher Scientific, Inc) (https://www.ncbi.nlm.nih.gov/geo/query/acc.cgi?acc=GSE24080). A dataset containing data from 1576 MM patients with 1q21 amplification (a combination of 4 independent cohorts; GSE4204, GSE 4452, GSE 4581, and GSE2658) was used for data validation. Additionally, for the enrichment analysis, the Gene Ontology enRIchmentanaLysis and visuaLizAtion (GOrilla) tool (http://cbl-gorilla.cs.technion.ac.il/) was used.

### Statistical analysis

Descriptive statistics, such as mean and standard deviation, are provided for variables. In this study, survival analysis of the patients was carried out using univariate and multivariate Cox regression methods with the help of R3.53 programming language and Kaplan–Meier graphics were created. R software “Survival” package (version 3.5.1; https://CRAN.R-project. Org/package = survival) was used to perform the survival analysis. As a result of the analysis, estimated hazard ratio (HR) and *P* values were obtained. HR obtained from the analysis showed that genes detected to be greater than 1 were “hazardous,” and those less than 1 were understood to have a “survival-triggering” characteristic. Additionally, Kaplan–Meier and Cox regression analyses were performed by using the intensity values of ARGs (41 genes) detected in patients with ABN. Gene ontology (GO) enrichment analysis was performed to identify the underlying biological process of different prognosis-related ARGs in the 2 patient groups (those with chromosome abnormalities and others). Statistical significance was accepted as *P* < 0.05.

In the Cox regression analyses, there are provided HRs, corresponding *P* values, and lower and upper 95% confidence intervals for the HRs.

Multicollinearity was checked for the independent variables that are used in the regression analysis. The bilateral (pairwise) correlation coefficient between all independent variables is computed using Eviews 4 software; developed by Quantitative Micro Software (QMS). As a result, pairwise correlations among the independent variables were quite low. On average, they amounted to 0.06 and none of them exceeded the threshold level of |0.5|, which is a commonly accepted level in the literature. Hence, it is possible to firmly state that no multicollinearity problem was observed in our analysis.

## Results

### Kaplan–Meier, univariate, and multivariate cox regression analyses results in MM patients

A summary of the study is provided in **Table 1**, and the sociodemographic data of the patients are shown in **Table 2**. The mean age of the patients was 57.04 ± 9.37 years (age range: 24.8–75.0 years). Among the cases, 88.6% were Caucasian and 60.4% female. CA were detected in 63.6% of the patients. During the treatment process, the Total Therapy 2 (TT2 – no thalidomide) protocol alone was applied to 60.9% of the patients. Furthermore, 39.05% of the patients received Total Therapy 3 (TT3 – bortezomib, thalidomide or lenalidomide, and dexamethasone) [12].

**Table 1.** Outline of the study

**Study dataset (548 MM patients, GSE24080)**	**Validation dataset 1576 MM patients, GSE4204, GSE4452, GSE4581, and GSE2658)**
Demographic properties of patients (**Table 2**) Survival Time According to Patients with/without Chromosomal Abnormality(**Table 3**) Univariate and multivariate Cox regression analyses and focus on the significant effect of ABN on survival of MM patients (**Figure 1, Tables 4** and **5**) Prognosis-related (survival-triggering or hazardous) ARG identification in patients with or without ABN (**Figures 2** and **3**) **Table 6** (for patients without ABN), **Table 7** (for patients with ABN) GOrilla Analysis (**Figure 4**)	Validation of prognosis-related ARGs in MM patients with ABN (only for 1q21 amplification) (**Table 8**)

ARG, autophagy-related genes; ABN, chromosomal abnormality; GOrilla, Gene Ontology enRIchmentanaLysis and visuaLizAtion; MM: multiple myeloma.

**Table 2.** Demographic properties in MM patients

**Parameters (n = 548)**	**n (%)**
Age (years); mean ± SD; 57.04 ± 9.37	
Gender	
Male	217 (39.59)
Female	331 (60.40)
ABN	
Yes	349 (63.68)
No	199 (36.31)
Treatment protocol	
TT2	334 (60.94)
TT3	214 (39.05)
Race	
Caucasian	486 (88.68)
Other	62 (11.31)

TT2 involves thalidomide treatment; TT3 involves bortezomib, thalidomide, and dexamethasone treatment. ABN, chromosomal abnormality; SD, standard deviation; MM: multiple myeloma; TT2, Total Therapy 2; TT3, Total Therapy 3.

**Figure 1.**
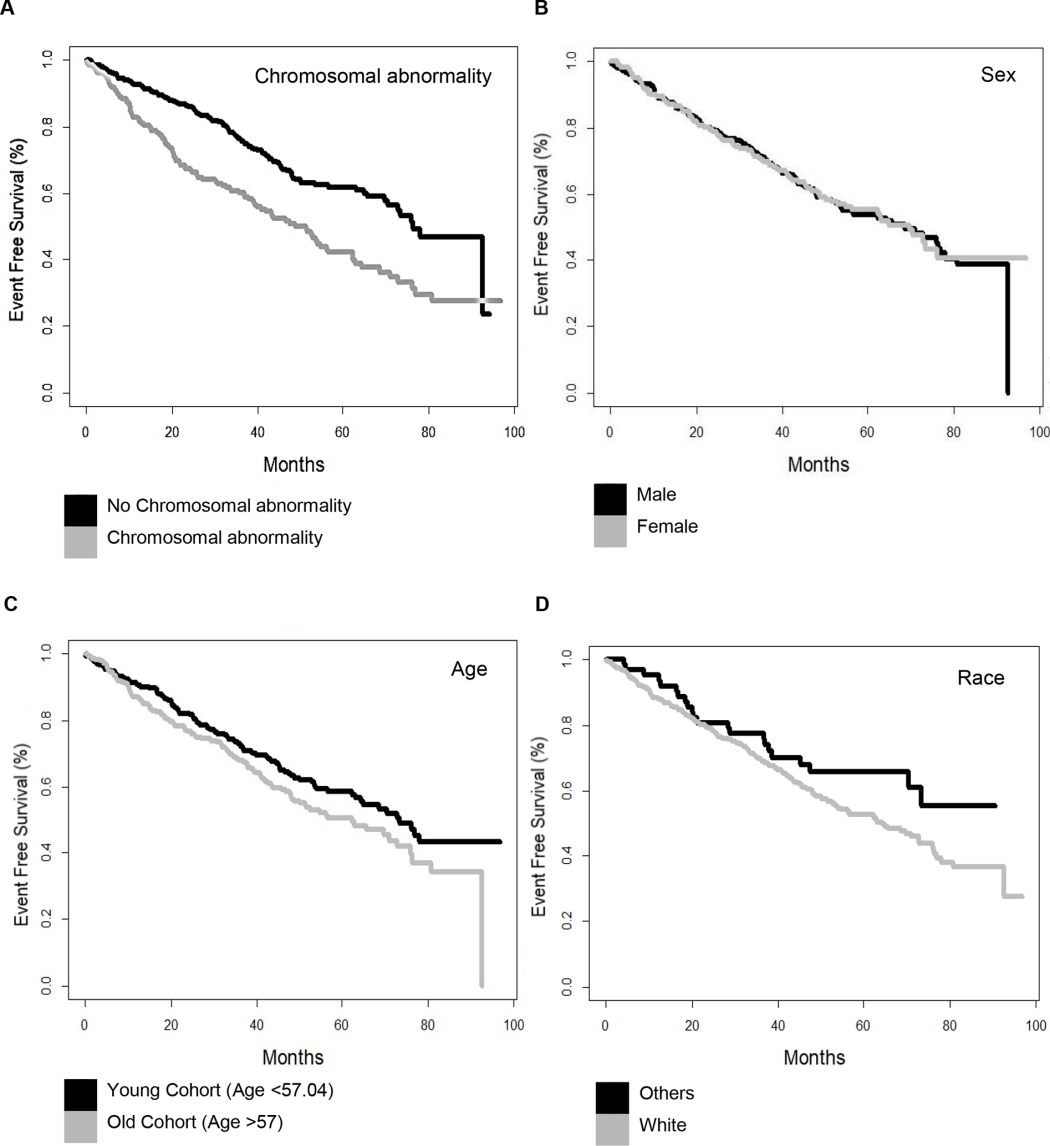

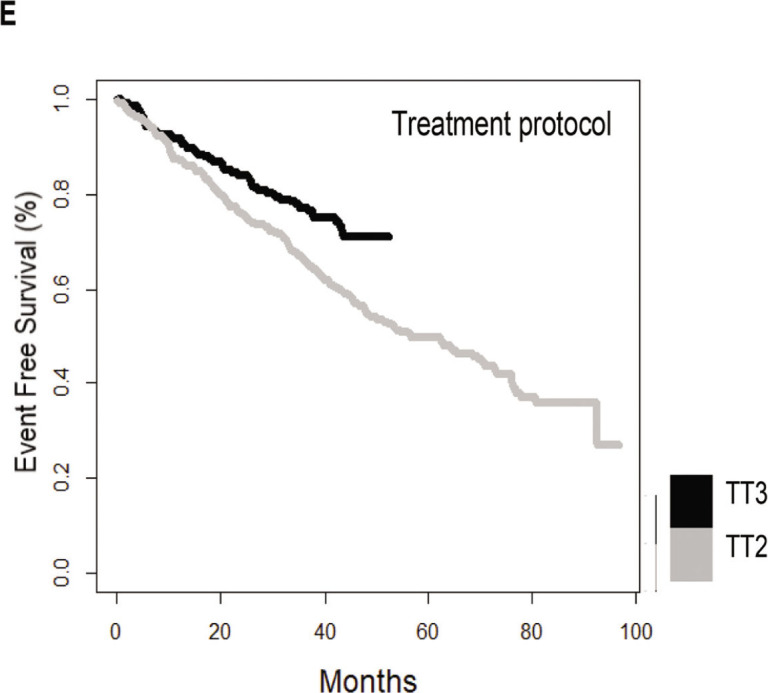
The significant effect of ABN on the EFS of MM patients. The effect of independent variables on both EFS of MM patients is shown using Kaplan–Meier survival plots as indicated in (**A**) ABN; EFS (**B**) sex and EFS; (**C**) age and EFS; (**D**) race and EFS; and (**E**) treatment protocol. ABN, chromosomal abnormality; EFS, event-free survival; MM: multiple myeloma.

In our study, the mean OS was found to be 48.2 months and the mean EFS was found to be 42.61 months. The OS was 42.7 months and the EFS was 37.6 months in patients with ABN (**Table 3**). It was observed that EFS times were lower in patients with ABN in MM (**Figure 1A**). On the other hand, the other factors were observed to be unimportant or weakly important (**Figures 1B–E**).

**Table 3.** Survival time according to patients with or without ABN

**Patients (months)**	**OS (mean ± SD)**	**EFS (mean ± SD)**
All patients (n = 548)	48.23 ± 22.36	42.61 ± 22.15
Patients with ABN (n = 349)	42.70 ± 24.79	37.63 ± 23.96
Patients without ABN (n = 199)	50.56 ± 20.50	44.93 ± 20.50

ABN, chromosomal abnormality; EFS, event-free survival; OS, overall survival; SD, standard deviation.

In the univariate and multivariate Cox regression analyses, it was observed that age and chromosomal anomaly significantly affected OS, while age, gender, and race variables did not significantly affect EFS. Additionally, ABN and treatment protocol variables were shown to significantly affect the EFS. HR of ABN and treatment protocol variables were found to be significantly above 1. The effect of the treatment protocol is important when EFS is considered as the dependent variable but becomes insignificant when OS is considered as the dependent variable. More importantly, the HR of the ABN is strongly significant in all situations, regardless of the dependent variable (EFS or OS) and regression type (univariate or multivariate). Moreover, patients with ABN have approximately twice the mortality rate than patients without ABN. Based on these results, we are able to infer that Cox regression analysis has enabled identification of the most, or even the only, effective variable among those deployed in the survival analysis, namely chromosomal abnormality (**Tables 4** and **5**).

**Table 4.** Univariate Cox regression results of OS and EFS analyses (n = 548)

**Dependent variables**	**EFS**				**OS**			
		
**Independent variables**	**HR**	**Lower 95% CI**	**Upper 95% CI**	***P***	**HR**	**Lower 95% CI**	**Upper 95% CI**	***P***
Age	1.0126	0.9989	1.027	0.0725	1.0222*	1.005	1.04	**0.0107**
Sex	0.9900	0.7652	1.281	0.9390	1.0018	0.7334	1.369	0.9910
Race	1.4538	0.9372	2.255	0.0948	1.0279	0.6367	1.657	0.9120
Treatment protocol	1.6409**	1.203	2.238	**0.0017**	1.1982	0.8316	1.727	0.3320
ABN	1.8009***	1.398	2.319	**0.0000519**	2.2054***	1.624	2.995	**0.0000408**

* *P* < 0.05;

** *P* < 0.01;

*** *P* < 0.001.

ABN, chromosomal abnormality; CI, confidence interval; EFS, event-free survival; HR, Hazard ratio; OS, overall survival.

**Table 5.** Multivariate Cox regression results of overall and event free survival analysis

**Dependent variables**	**EFS**				**OS**			
		
**Independent variables**	**Hazard ratio**	**Lower 95% C.I**	**Upper 95% C.I**	***P***	**Hazard ratio**	**Lower 95% C.I**	**Upper 95% C.I**	***P***
Age	1.0113	0.9971	1.026	0.1202	**1.0234**	1.0057	1.041	**0.0093**
Sex	0.9962	0.7689	1.291	0.9773	1.0308	0.7537	1.41	0.8489
Race	1.4049	0.8969	2.201	0.1375	0.9012	0.5515	1.473	0.6781
Treatment protocol	1.6626**	1.2168	2.272	**0.0014**	1.2065	0.8357	1.742	0.3163
Chromosomal abnormality	1.7627***	1.3676	2.272	**0.00012**	2.1790***	1.6038	2.961	**0.0000633**

* *P* < 0.05;

** *P* < 0.01;

*** *P* < 0.001.

ABN, chromosomal abnormality.

### Kaplan–Meier, univariate, and multivariate cox regression analyses results according to ARG in MM patients with and without chromosomal abnormalities

In our study, many ARGs that significantly affect the prognosis of MM patients were detected (n = 548). In the first stage, 31 ARGs were found to have a significant effect on MM prognosis based on univariate and multivariate Cox regression analyses results, including intensity values of 222 ARGs and other independent variables (age, gender, race, treatment protocol, and ABN) (data not shown).

In the second stage, ARGs that affect the survival of patient groups with and without ABN were examined separately. The data of the patients in the group without ABN (n = 199) are presented in **Table 6**. A total of 13 statistically significant ARGs were detected, and the HR value of only 1 of these 13 genes (*ATG16L2*) was above 1. On the other hand, 41 ARGs were found significant in MM patients with chromosomal anomalies (n = 349) (*P* < 0.05, **Table 7**). The HRs of 20 of these 41 genes (*ARNT, ATIC, BIRC5, CAPN10, CASP3, CDKN2A, EIF2S1, EIF4EBP1, EIF4G1, FADD, FKBP1A, GNAI3, HDAC6, HGS, HSP90AB1, MAPK1, MBTPS2, PARP1, TSC2,* and *WDFY3*) were above 1.

**Table 6.** Univariate and multivariate Cox regression results of prognosis-associated ARG in MM patients without ABN

**Univariate and multivariate Cox regression results of prognosis-associated ARG in MM patients without ABNGene**	**Univariate Cox regression**				**Multivariate Cox regression**			
	
	**HR**	**Lower 95% CI**	**Upper 95% CI**	***P***	**HR**	**Lower 95% CI**	**Upper 95% CI**	***P***
*APOL1*	0.80962*	0.6862	0.9553	0.0124	0.828576*	0.6996	0.9813	0.02935
*ARSA*	0.83103**	0.7354	0.9391	0.003	0.845735**	0.7462	0.9586	0.00873
*ATG16L2*	1.2505*	1.013	1.543	0.0374	1.30139*	1.0507	1.612	0.01583
*ATG9A*	0.6562***	0.5114	0.8419	0.000923	0.705951**	0.5506	0.9051	0.00603
*ATG101*	0.6862**	0.5156	0.9132	0.0098	0.703135*	0.5146	0.9608	0.02703
*CDKN1A*	0.7468*	0.5849	0.9536	0.0192	0.74999*	0.5830	0.9648	0.02519
*DNAJB1*	0.7074*	0.5316	0.9414	0.0176	0.72321*	0.5419	0.9653	0.02781
*DRAM1*	0.7561*	0.5756	0.9932	0.0446	0.74669*	0.5729	0.9733	0.030754
*FKBP1B*	0.82008**	0.7218	0.9318	0.00233	0.830138**	0.7293	0.9449	0.00483
*FOXO3*	0.7109**	0.5585	0.905	0.00559	0.70415**	0.5516	0.899	0.00488
*MAPK9*	0.6577*	0.4565	0.9474	0.0244	0.649662*	0.4491	0.9398	0.022037
*NCKAP1*	0.8088***	0.7198	0.9089	0.000362	0.82788**	0.7360	0.9312	0.00164
*RAB24*	0.7536*	0.5958	0.953	0.0182	0.727826**	0.5723	0.9256	0.009599

* *P* < 0.05;

** *P* < 0.01;

*** *P* < 0.001.

ABN, chromosomal abnormality; ARG, autophagy-related genes; CI, confidence interval; HR, Hazard ratio; MM, Multiple myeloma

**Table 7.** Univariate and Multivariate Cox regression results of prognosis-associated ARG in MM patients with ABN

**Gene**	**Univariate Cox regression**				**Multivariate Cox regression**			
	
	**HR**	**Lower 95% CI**	**Upper 95% CI**	***P***	**HR**	**Lower 95% CI**	**Upper 95% CI**	***P***
ARNT	1.5304*	1.064	2.202	0.0219	1.498746*	1.0238	2.194	0.0374
ATG4B	0.5552*	0.3172	0.972	0.0395	0.554451*	0.3167	0.9708	0.0391
ATG4D	0.5521***	0.3891	0.7833	0.0008	0.568641**	0.3919	0.825	0.00295
ATIC	1.8684**	1.236	2.824	0.00302	1.857592**	1.2147	2.841	0.00427
BIRC5	1.30634***	1.163	1.467	6.22e–06	1.343899***	1.1901	1.518	1.87e–06
CAPN10	1.21016*	1.008	1.453	0.041	1.26361*	1.0470	1.525	0.0147
CASP3	1.4334*	1.013	2.029	0.0422	1.505861*	1.0472	2.165	0.0272
CDKN1A	0.7103**	0.5645	0.8938	0.00353	0.726627**	0.5787	0.9124	0.00598
CDKN2A	1.4842*	1.056	2.085	0.0228	1.54526*	1.0525	2.269	0.0263
CXCR4	0.73568***	0.6289	0.8606	0.000125	0.765047**	0.6486	0.9025	0.00148
DNAJB9	0.6088*	0.389	0.9529	0.0299	0.593613*	0.3766	0.9356	0.0246
EIF2S1	1.6981**	1.156	2.494	0.00692	1.79448**	1.1872	2.712	0.00553
EIF4EBP1	1.34577***	1.128	1.605	0.000955	1.312952**	1.1006	1.566	0.00249
EIF4G1	2.3264***	1.493	3.626	0.000193	2.206738***	1.3948	3.491	0.000721
FADD	1.5694*	1.063	2.317	0.0234	1.61684*	1.0894	2.400	0.01708
FKBP1A	1.8452**	1.187	2.868	0.00647	1.88003**	1.1997	2.946	0.00588
FKBP1B	0.82577**	0.7243	0.9414	0.0042	0.851602*	0.7454	0.973	0.0181
FOXO1	0.6549*	0.4739	0.9051	0.0103	0.645133**	0.4668	0.8917	0.00794
FOXO3	0.7363*	0.5657	0.9583	0.0228	0.72246*	0.5501	0.9489	0.01942
GABARAP	0.4739**	0.2896	0.7753	0.00295	0.464234**	0.2809	0.7672	0.00276
GNAI3	1.5955*	1.082	2.353	0.0184	1.542438*	1.0415	2.284	0.0305
HDAC6	1.7322*	1.059	2.834	0.0288	1.645421*	1.0051	2.694	0.0477
HGS	1.8098*	1.14	2.873	0.0119	1.696411*	1.0619	2.710	0.027
HSP90AB1	1.6281*	1.088	2.436	0.0178	1.594093*	1.0567	2.405	0.0262
IL24	0.82369*	0.6869	0.9877	0.0363	0.795624*	0.6579	0.9622	0.0184
IRGM	0.81103*	0.6737	0.9763	0.0269	0.823283*	0.6834	0.9919	0.0408
ITGA6	0.67844***	0.5709	0.8063	1.06e–05	0.686741**	0.5770	0.8174	2.35e–05
ATG13	0.4507***	0.2866	0.7087	0.000558	0.425973**	0.2721	0.6669	0.00019
LAMP2	0.7123*	0.5495	0.9234	0.0104	0.73084*	0.5619	0.9505	0.0193
MAPK1	1.496*	1.027	2.18	0.0359	1.481801*	1.0079	2.179	0.0455
MBTPS2	1.445*	1.039	2.009	0.0287	1.435077*	1.0182	2.023	0.0391
NCKAP1	0.8715*	0.7823	0.9709	0.0126	0.8927*	0.7987	0.9978	0.0456
PARP1	1.8204***	1.286	2.577	0.000729	1.938906***	0.8540	2.094	0.000271
PRKCD	0.7481*	0.5748	0.9735	0.0308	0.751456*	0.5741	0.9837	0.03755
SIRT2	0.5835**	0.4089	0.8327	0.00298	0.662074*	0.4521	0.9696	0.0341
TNFSF10	0.88013*	0.7841	0.988	0.0304	0.87052*	0.7759	0.9767	0.01824
TP53	0.7408**	0.6079	0.9029	0.00296	0.726943**	0.5947	0.8887	0.00186
TSC2	1.5559*	1.099	2.202	0.0126	1.4891644*	1.0499	2.112	0.0255
VAMP3	0.571***	0.4168	0.7823	0.000485	0.596231**	0.4317	0.8235	0.0017
WDFY3	1.1406*	1.017	1.28	0.025	1.132911*	1.0127	1.267	0.0293
WDR45L	0.5495*	0.3283	0.9198	0.0227	0.57379*	0.3396	0.9696	0.0379

* *P* < 0.05;

** *P* < 0.01;

*** *P* < 0.001.

ABN, chromosomal abnormality; ARG, autophagy-related genes; CI, confidence interval; HR, Hazard ratio; MM, Multiple myeloma.

According to these results, it is seen that 20 different genes in patients with ABN compared to patients without ABN are “hazardous” in terms of the prognosis of MM. In addition to these findings, only 4 common ARGs (*CDKN1A, FKBP1B, FOXO3,* and *NCKAP1*) have been detected such that they significantly affect both the ABN and non-ABN patient groups. The HRs of these genes are below 1. Therefore, it is understood that it is necessary to analyze the patients individually and understand the presence of ABN in their genetic history to avoid misleading results, which could in turn have an adverse impact on the course of treatment decided upon.

**Figure 2.**
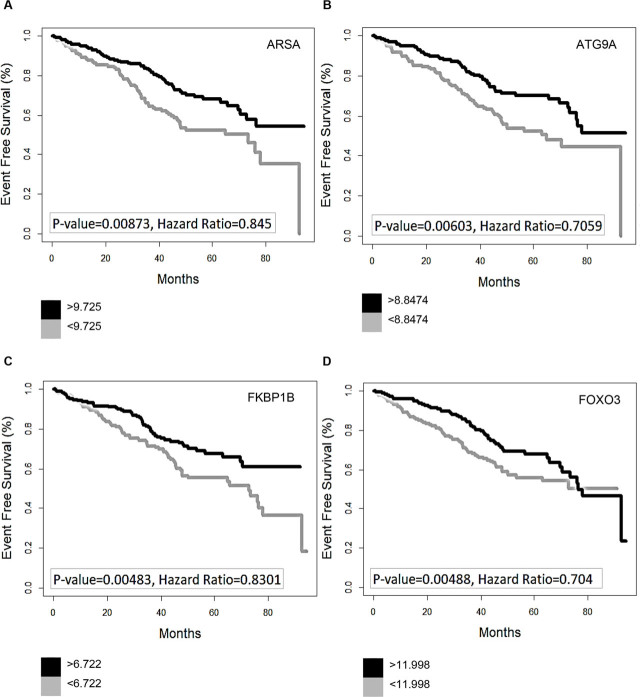

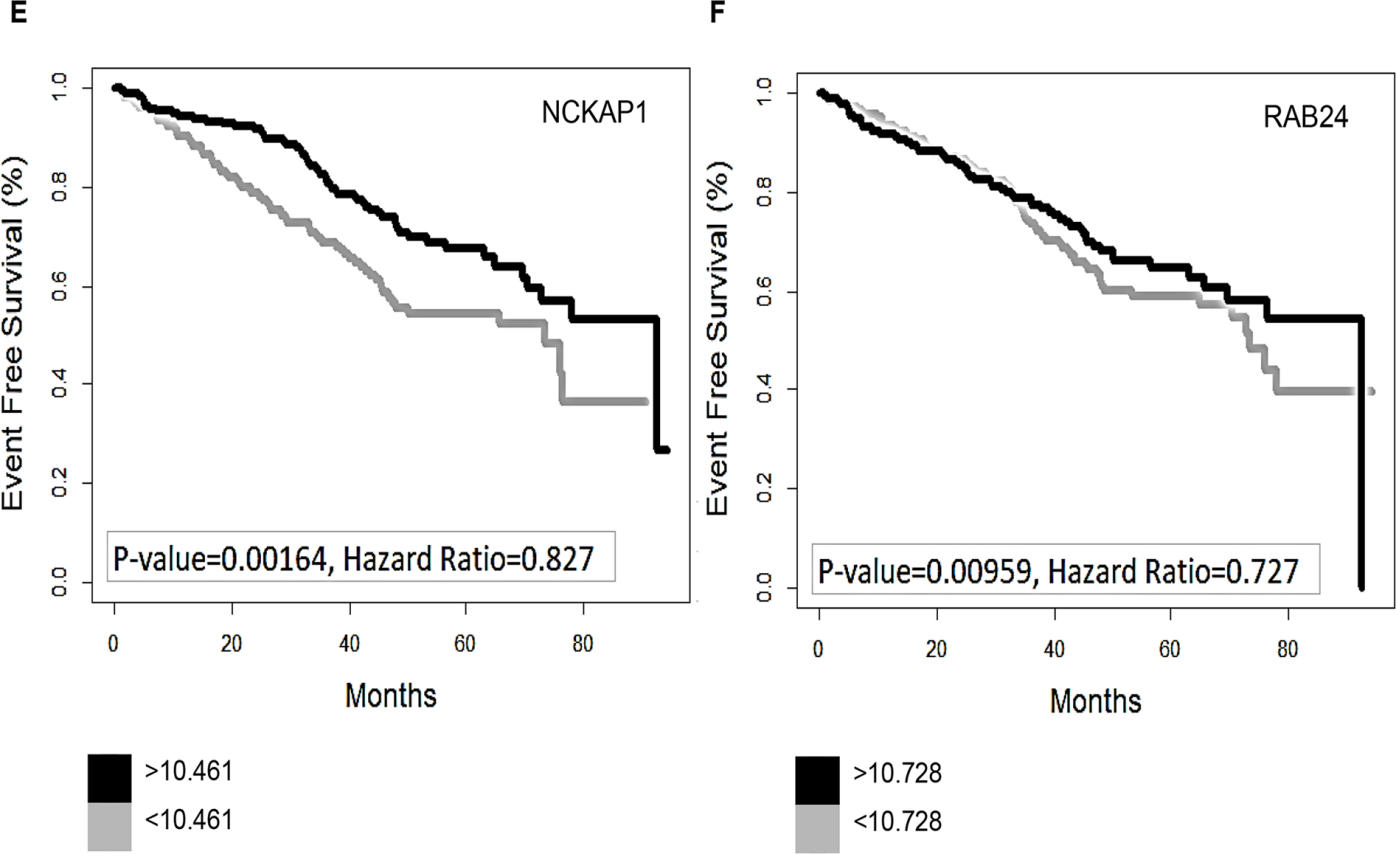
Survival analysis of selected ARGs in MM patients without ABN; Kaplan–Meier plots of 6 prognosis-related ARGs in MM patients without ABN. (**A**) *ARSA*, (**B**) *ATG9A,* (**C**) *FKBP1B*, (**D**) *FOXO3*, **(E)** *NCKAP1*, and (**F**) *RAB24*. *ARSA*, arylsulfatase A; *ATG9A*, *ATG9* autophagy-related 9 homolog A (*Saccharomyces cerevisiae*); *FKBP1B, FK506* binding protein 1B, 12.6 kDa; *FOXO3*, forkhead box O3; *NCKAP1*, NCK-associated protein 1; *RAB24*, *RAB24*, member RAS oncogene family. ABN, chromosomal abnormality; ARGs, autophagy-related genes.

**Figure 3.**
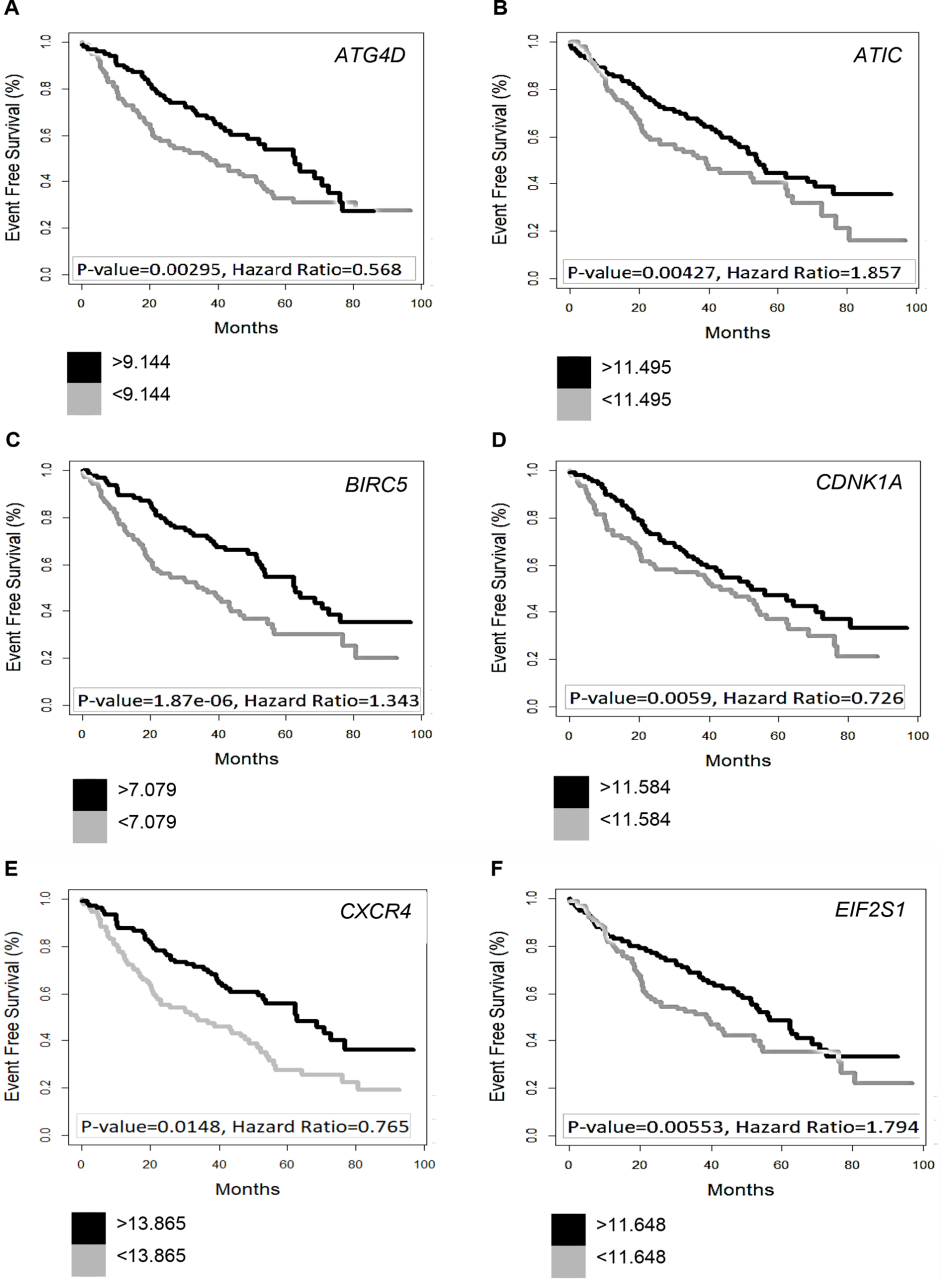

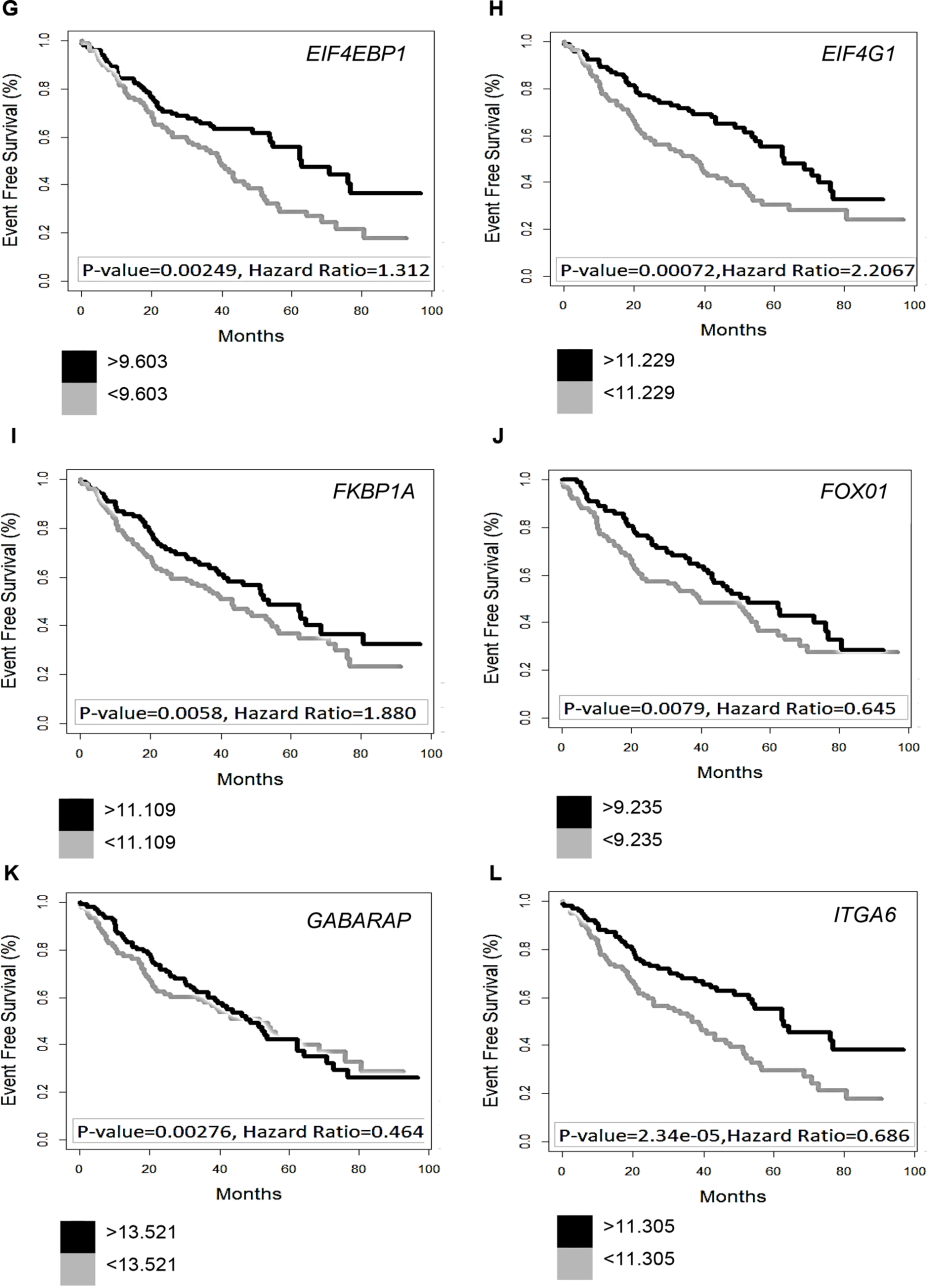

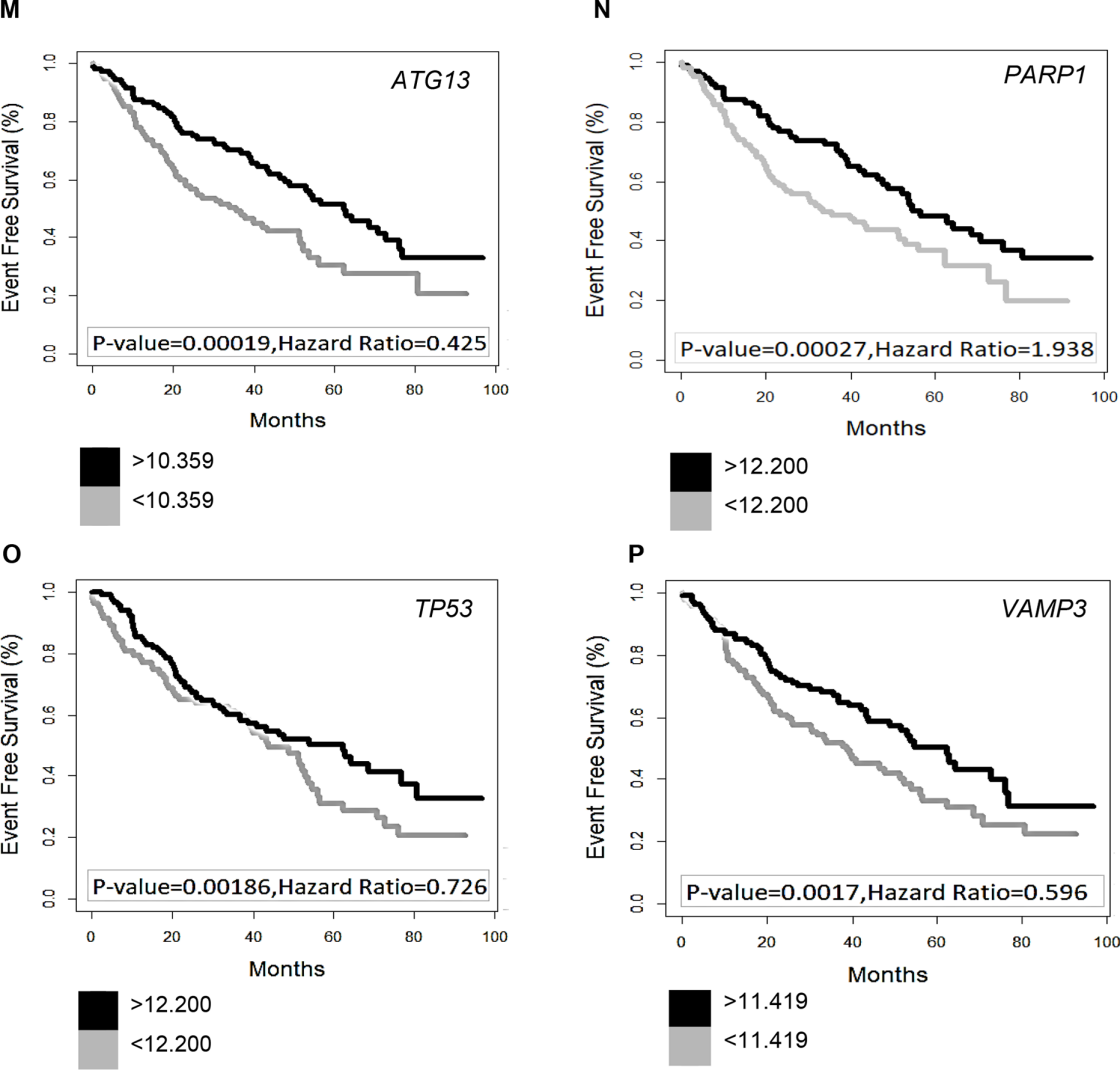
Survival analysis of selected ARGs in MM patients with ABN; Kaplan–Meier plots of 6 prognosis-related ARGs in MM patients with ABN. (**A**) *ATG4D* (*S. cerevisiae*); (**B**) *ATIC*; (**C**) *BIRC5*; (**D**) *CDKN1A* (p21, Cip1); (**E**) *CXCR4*; (**F**) *EIF2S1*, 35 kDa; (**G**) *EIF4EBP1*; (**H**) *EIF4G1*; (**I**) *FKBP1A*, 12 kDa; (**J**) *FOXO1*; (**K**) *GABARAP*; (**L**) *ITGA6*; (**M**) *ATG13*, *KIAA0652*; (**N**) *PARP1*; (**O**) *TP53*; (**P**) *VAMP3* (cellubrevin). ABN, chromosomal abnormality; ARGs, autophagy-related genes; *ATG4D*, TG4 autophagy-related 4 homolog D; *ATG13*, Autophagy-Related Protein 13; *ATIC*, 5-aminoimidazole-4-carboxamide ribonucleotide formyltransferase–IMP cyclohydrolase; *BIRC5*, baculoviral IAP repeat-containing 5; *CDKN1A*, cyclin-dependent kinase inhibitor 1A; *CXCR4*, chemokine (C-X-C motif) receptor 4; *EIF2S1*, eukaryotic translation initiation factor 2, subunit 1 alpha; *EIF4EBP1*, eukaryotic translation initiation factor 4E binding protein 1; *EIF4G1*, eukaryotic translation initiation factor 4 gamma, 1; *FKBP1A*, FK506 binding protein 1A; *FOXO1*, forkhead box O1; *GABARAP*, GABA(A) receptor-associated protein; ITGA6, integrin, alpha 6; MM: multiple myeloma; *PARP1*, poly (ADP-ribose) polymerase 1; *TP53*, tumor protein p53; *VAMP3*, vesicle-associated membrane protein 3.

The effects of gene intensities of the most important ARGs (*P* < 0.001) are shown in Kaplan–Meier plots (**Figure 2**, no ABN; and **Figure 3**, ABN). It is demonstrated clearly that prognosis-related ARGs are quite different across with- and without-ABN groups and that these genes are very influential on the survival of patients.

### Functional GO enrichment analysis

A functional gene enrichment analysis was performed to determine the biological significance of the study. The ARGs determined according to the results using the data of patients without ABN were associated with the “biological process” themes. In the “biological process,” general phenomena such as “protein localization” and “peptide and amide transport” processes were associated with ARGs (data not shown).

**Figure 4.**
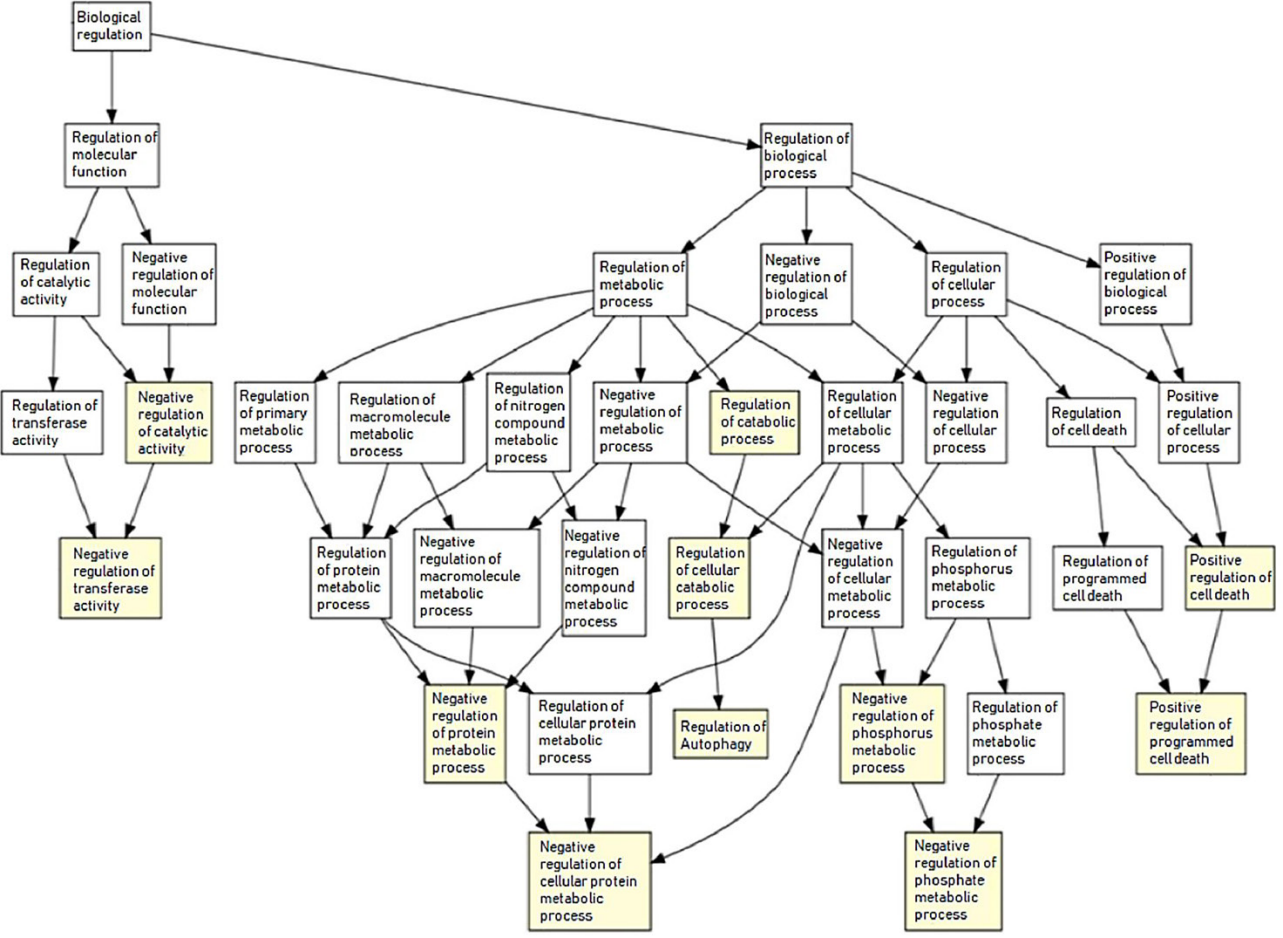
GOrilla biological process in MM patients with ABN. ABN, chromosomal abnormality; GOrilla, Gene Ontology enRIchmentanaLysis and visuaLizAtion; MM: multiple myeloma.

In **Figure 4**, the results of the analyses using the data of patients with ABN are presented. Accordingly, the results focused on more specific “biological processes,” such as regulation of “programmed cell death” and “regulation of autophagy.” Exceptionally, the concepts of “regulation of phosphate metabolic process” and “regulation of transferase activity” were also significantly associated with ARGs of patients with ABN.

### Validation of prognosis-related ARGs

Since ABN is the main focus of the study, it is important to verify the prognosis-related ARGs in our study in MM patients with ABN using other datasets. Other datasets including both ARGs and the presence and absence of ABN in MM patients were thoroughly investigated, but no suitable dataset was detected. Therefore, datasets containing data of 1576 MM patients with 1q21 amplification, which are known to be very important in prognosis in the literature, were used. Cox regression results for the confirmation of ARGs associated with prognosis are given in **Table 8**. A total of 29 ARGs were found to significantly affect the prognosis of 1q21 amplified MM patients. According to the results, most of the prognosis-related ARGs detected by the study dataset in our research were confirmed (29/41; 13 “hazardous” genes with HR >1), which largely confirms the consistency of our results.

**Table 8.** Validation of prognosis-related ARGs in MM patients with ABN

**Gene**	**Univariate Cox regression**			

	**HR**	**Lower 95% CI**	**Upper 95% CI**	***P***
ARNT	0.78874**	0.6672	0.9324	0.00543
ATG4B	2.1821***	1.51	3.154	3.29e–05
ATG4D	0.2839***	0.1907	0.4226	5.56e–10
ATIC	3.5622***	2.485	5.107	4.72e–12
CAPN10	0.4837***	0.3665	0.6383	2.87e–07
CASP3	0.84905*	0.7409	0.973	0.0186
CDKN1A	0.76380***	0.6658	0.8762	0.00012
CDKN2A	1.51399***	1.356	1.69	1.55e–13
CXCR4	0.5576***	0.4568	0.6807	9.5e–09
DNAJB9	1.7560**	1.193	2.584	0.00427
EIF4EBP1	0.81096***	0.726	0.9059	0.000207
EIF4G1	0.6512**	0.4911	0.8633	0.00287
FADD	1.6059**	1.179	2.188	0.00268
FKBP1A	0.3130***	0.2093	0.4679	1.5e–08
FKBP1B	0.6616***	0.5228	0.8374	0.000589
FOXO1	0.3332***	0.24	0.4626	5.25e–11
FOXO3	0.7879*	0.6265	0.9908	0.0415
GNAI3	0.79854**	0.6732	0.9472	0.00981
HGS	0.70614***	0.6254	0.7973	1.98e–08
HSP90AB1	0.76746***	0.6908	0.8526	8.12e–07
IL24	2.5083***	1.675	3.756	7.98e–06
IRGM	2.3205***	1.664	3.236	7.07e–07
ITGA6	4.0435***	2.847	5.744	6.11e–15
ATG13	0.3571***	0.2254	0.5658	1.16e–05
LAMP2	2.4952***	1.774	3.509	1.48e–07
MAPK1	2.0933***	1.395	3.142	0.000363
TNFSF10	1.2571*	1.021	1.548	0.0314
TP53	1.25520*	1.038	1.518	0.019
TSC2	1.4152*	1.04	1.925	0.027

* *P* < 0.05;

** *P* < 0.01;

*** *P* < 0.001.

ABN, chromosomal abnormality; ARGs, autophagy-related genes; CI, confidence interval; HR, Hazard ratio; MM, Multiple myeloma

## Discussion

The present study undertakes an investigation into the role of ABN in the survival analysis of MM patients. Our findings address 2 major conclusions. First, it is clearly shown that ABN is the most severe factor causing mortality (among other factors such as sex, age, treatment protocol, and race). Second, it is evident that the patients with ABN have radically different prognosis-related novel ARGs (mostly hazardous ones) compared with the patients without ABN.

The clinical effect of ABN on poor patient outcome has been noted in various cancers including MM [13,14,15,16,17]. To develop more effective cancer treatments, it is extremely important to determine the stage of cancer as a pathological staging based on the tumor-node-metastasis (TNM) classification system [18]. However, this system does not show the biological background and molecular basis of cancers. Therefore, it provides insufficient information about the genetic basis of malignancy and the prediction of prognosis of the disease. Recently, it has become a tradition to trace individual cancer-related genes to enable the targeting of molecular mechanisms and the detection of strong biomarkers at the gene level. In this context, genes related to autophagy have been screened using high-throughput expression data and valuable statistical tools in recent years. In this way, identified candidate ARGs that can affect the prognosis of different cancers, including cancers such as MM, were targeted [18,19,20,21,22,23]. Zhu et al. [24] demonstrated the prognostic value of 16 ARGs in 559 patients with MM and developed a model based on the risk score. However, the dataset used in the study conducted by Zhu et al. [24] included all patients regardless of their CA. The role of ABN in a survival analysis of MM patients was investigated in our study. Prognostic ARGs (prognostic ARG signature) were attempted to be determined for patient groups with and without chromosomal anomalies. As a result of the analyses performed, it was clearly shown in our study that ABN was the most serious factor that causes death (among other factors such as gender, age, treatment protocol, and race). Second, it was clear that patients with ABN (41 different ARGs) had different prognosis-related ARGs (mostly dangerous ones) than patients without ABN (13 different ARGs). These hazardous and survival-triggering ARGs (corresponding to MM patients with and without ABN, respectively), which differ according to the genetic background of the patients, can be reliable and promising targets to eradicate MM cancer and increase the survival of patients.

These results provide very important implications for cancer treatment outcomes and pharmacological research and applications. The results of the studies in the literature showed that the presence of ABN may lead to therapeutic vulnerability in cancers [25, 26]. According to the results of these studies, it can be concluded that differently designed cancer treatments can change the fate of cancer prognosis. Therefore, when starting a treatment strategy, investigating the presence or absence of ABN in the primary stage can increase the success of the treatment. Similarly, in addition to classical cancer staging procedures, the European Myeloma Network recommends subgrouping patients with MM based on their cytogenetic history and ABN prior to optimization of anticancer drug combinations [27]. It is promising to subgroup MM patients using a combination of the International Staging System (ISS) and ABN to have higher survival rates, according to the results of an international myeloma working group [28]. Therefore, in our study, 548 MM patients from the MAQC-II project, whose gene intensity data with GEO GSE24080 accession number are available, were divided into 2 groups according to their genetic history (MM patients with and without ABN). This strategy will differ significantly in 2 different situations. On the one hand, if there is a ABN, most prognosis-related ARGs can be downregulated at the gene level, as they are often dangerous, and can contribute positively to treatment. On the other hand, in patients without ABN, cancer treatment agents can be selected in such a way that survival-triggering ARGs can be upregulated, and those with hazardous ones can be designed to be downregulated. These applications should be tested with laboratory-based clinical studies and advanced bioinformatics analyses, further studies should be carried out on drug interactions and gene interactions with each other, and gene therapies should be designed according to these studies. However, in our study, the effects of specific types of ABN (such as mutations, cytogenetic changes, translocations, etc.) on survival times and rates of MM patients have not been searched since the data with the accession number GSE24080 do not include these detailed elements of information.

Although different research groups have tried to focus on the chromosome abnormalities of MM patients to develop better therapeutic strategies targeting cells based on their genetic background, this has not been emphasized enough in the literature [29, 30]. First, there is a limited data set based on genetic background and ABN including cancer patient gene densities. For instance, to our knowledge, MM patient gene intensity data on specific types of ABN and mutations have also not been available. Therefore, a purposeful designing of the data mining and data collection stages can predominantly ensure the reliability of the results obtained from bioinformatics analysis. Otherwise, current survival analysis and laboratory studies can lead to misleading results. Additionally, it can be very important to be aware of the diversity of cancer-related genes (e.g., ARGs) in pharmaceutical industry applications and drug development steps to avoid wasting time and investment. This can be achieved by using reliable patient gene density data for bioinformatics analysis.

In our study, the role of autophagy-dependent genes in cancer treatment, and therefore, the biological processes associated with autophagy, were shown to play a key role in cancer treatment, at least in MM cancer. The literature findings also support our studies. Ongoing lab-based research has shown that gene-level targeting with anticancer drugs in MM is a promising strategy for suppressing autophagy and related pathways as well as eradicating MM cancer [31, 32].

In our study, due to the lack of data of patients with other ABN, our results were confirmed only by the data of 1576 MM patients with 1q21 amplification. Despite the lack of data, the biological significance of our verification was high. In the literature, 1q21 amplification was associated with poor prognosis and shorter survival of MM patients [33]. In parallel with these results, it was shown in our study that 29 of 41 ARGs found to affect the prognosis of patients with ABN were associated with prognosis. It has also been ascertained in the literature that the combination of 1q21 amplification with other ABN would lead to an even shorter survival time [33]. A greater number of ARGs (more than 29) could have been confirmed in our study if data pertaining to other chromosomal abnormalities had been available.

In parallel with the results of functional GO enrichment analysis, in patients without ABN, general “biological processes” such as “protein localization” and “peptide and amide transport” came to the fore. However, in cases of ABN, enriched ARGs were mainly related to specific pathways such as programmed cell death, positive regulation of stress conditions (such as starvation, reactive oxygen species (ROS), organonitrogen compounds), negative regulation of transferase activity, phosphate metabolic process, and cellular protein metabolic process. In other words, GOrilla highlights the enrichment in ARGs related to cell death and stress response pathways in patients with ABN. Accordingly, according to the results of GOrilla clustering, targeting specific ARGs related to programmed cell death by autophagy by triggering stress conditions may be an effective way to combat MM.

Despite recent therapeutic advances, MM cancer is not yet effectively treatable. For example, the IL-3 cytokine is required for B cells to survive, and in our previous in vitro and in vivo studies, IL-3 withdrawal was shown to induce autophagic cell death and subsequent apoptosis in pro-B cells. Following IL-3 withdrawal, cancer cells could be removed after phagocytosis by resident macrophages [34]. There are other studies targeting autophagic cell death in MM cells. For example, Lamy et al. [35] demonstrated that caspase-10 is essential for the survival of all MM cell lines and that the vital balance between survival and cell death can be disturbed and driven in favor of autophagic cell death. Another study concluded that iron deprivation and hence higher cellular and mitochondrial ROS levels could lead to autophagic cell death in MM cells [36]. These results are consistent with our current findings that it may be possible to eradicate MM as a clonal B cell malignancy by inducing excessive starvation or other stress conditions and thus manipulating specific ARGs and thereby inducing autophagic cell death. Similarly, in another study, another type of cancer cells, human breast cancer cells (MCF-7), were induced to die by autophagy through steroid hormone depletion and tamoxifen therapy. These autophagic dying cells were shown to be phagocytosed by human macrophages, and clearance of these cells was achieved by inflammasome activation [37]. As a result, we believe that further research into cancer treatment can focus on cancer treatment from this perspective and increase the chances of success in treatment.

To sum up, ARGs can be involved in clinical practice and treatment strategies in MM patients with ABN. Based on the results we obtained with gene ontology analysis (GOrilla analysis), it may be possible to use ARGs detected in MM patients with ABN as target genes in anticancer treatments. For instance, autophagy is involved in cancer treatment according to the stage of the cancer. Therefore, autophagy can be either triggered or blocked to combat MM cancer cells with ABN according to their stages (early or late stage). Additionally, autophagic cell death can even be triggered by targeting related ARGs in MM patients with ABN.

## Conclusion

Our study has some limitations. First, there is a lack of data including gene intensity values of patients with ABN as well as genes linked to autophagy. The results were validated with this dataset, since only data from 1q21 amplified MM patients were available. Second, the prognostic value and clinical significance of these ARGs must be tested using further laboratory-based research. Third, the interaction between the genes identified for autophagy and each ABN should be investigated. In addition to our gene enrichment results, the question of why some genes affect survival in the presence of ABN should be answered by using clinical samples with laboratory-based research.

Consequently, in this study, the prognostic model was constructed in such a way that the status of ARG genes changed because of the presence or absence of ABN. Classification of MM patients according to their ABN can provide us with a more personalized and efficient cancer treatment.
